# Mutations in epigenetic regulators are involved in acute lymphoblastic leukemia relapse following allogeneic hematopoietic stem cell transplantation

**DOI:** 10.18632/oncotarget.6259

**Published:** 2015-10-30

**Authors:** Haowen Xiao, Li-Mengmeng Wang, Yi Luo, Xiaoyu Lai, Caihua Li, Jimin Shi, Yamin Tan, Shan Fu, Yebo Wang, Ni Zhu, Jingsong He, Weiyan Zheng, Xiaohong Yu, Zhen Cai, He Huang

**Affiliations:** ^1^ Bone Marrow Transplantation Center, The First Affiliated Hospital, Zhejiang University School of Medicine, Hangzhou, P R China; ^2^ Department of Hematology, Guangzhou General Hospital of Guangzhou Military Command (Guangzhou Liuhuaqiao Hospital), Guangzhou, P R China; ^3^ Center for Genetic and Genomic Analysis, Genesky Biotechnologies Inc., Shanghai, P R China

**Keywords:** acute lymphoblastic leukemia, relapse, allogeneic hematopoietic stem cell transplantation, mutation, epigenetic regulators

## Abstract

Although steady improvements to chemotherapeutic treatments has helped cure 80% of childhood acute lymphoblastic leukemia (ALL) cases, chemotherapy has proven to be less effective in treating the majority of adult patients, leaving allogeneic hematopoietic stem cell transplantation (allo-HSCT) as the primary adult treatment option. Nevertheless relapse are the leading cause of death following allo-HSCT. The genetic pathogenesis of relapse following allo-HSCT in Philadelphia chromosome- negative ALL (Ph^−^ ALL) remains unexplored. We performed longitudinal whole-exome sequencing analysis in three adult patients with Ph^−^ B-cell ALL (Ph^−^ B-ALL) on samples collected from diagnosis to relapse after allo-HSCT. Based on these data, we performed target gene sequencing on 23 selected genes in 58 adult patients undergoing allo-HSCT with Ph^−^ B-ALL. Our results revealed a significant enrichment of mutations in epigenetic regulators from relapsed samples, with recurrent somatic mutations in *SETD2*, *CREBBP*, *KDM6A* and *NR3C1*. The relapsed samples were also enriched in signaling factor mutations, including *KRAS*, *PTPN21*, *MYC* and *USP54*. Furthermore, we are the first to reveal the clonal evolution patterns during leukemia relapse after allo-HSCT. Cells present in relapsed specimens were genetically related to the diagnosed tumor, these cells therefore arose from either an existing subclone that was not eradicated by allo-HSCT therapy, or from the same progenitor that acquired new mutations. In some cases, however, it is possible that leukemia recurrence following allo-HSCT could result from a secondary malignancy with a distinct set of mutations. We identified novel genetic causes of leukemia relapse after allo-HSCT using the largest generated data set to date from adult patients with Ph^−^ B-ALL.

## INTRODUCTION

Relapsed hematologic malignancies are the leading cause of death following allogeneic hematopoietic stem cell transplantation (allo-HSCT) [1]. The prognosis is particularly severe in adult acute lymphoblastic leukemia (ALL) patients. Although steady improvements to chemotherapeutic treatments has helped cure 80% of childhood ALL cases, chemotherapy has proven to be less effective in treating the majority of adult patients, leaving allo-HSCT as the primary adult treatment option [2]. Nevertheless, 20%–40% of patients experiences relapse following allo-HSCT in the first complete remission (CR), and relapse incidences increase to 30%–50% during the second CR, resulting in an overall relapsed patient survival rate of less than 10% [3–7].

Several adverse genetic alterations, including rearrangement of the myeloid- lymphoid or mixed-lineage leukemia genes and Philadelphia (Ph) chromosomes, are ultimately responsible for ALL treatment failure and relapse [8]. However, many Ph chromosome-negative (Ph^−^) ALL patients that have a normal karyotype and lack documented risk factors may experience relapse as well. These particular cases currently lack genomic and genetic biomarkers to assist in their prognosis and treatment. Furthermore, there is a lack of comprehensive and dynamic analyses that characterize the genetic alterations from diagnosis to relapse for adult ALL patients following allo-HSCT treatment. Notably, this is in direct contrast to traditional chemotherapies. Relapse occurrence after allo-HSCT relies on two processes. Firstly, malignant cells must survive the intensive chemotherapy and/or radiotherapy conditioning regimen that precedes allo-HSCT. Secondly, following the allo-HSCT procedure, cells must survive the effects of the graft-versus-leukemia reaction [9]. We therefore hypothesized that critical genetic factors in Ph^−^ ALL patients confer leukemic cells with the ability to withstand multiple selective pressures, thus allowing them to expand and ultimately promote relapse post-HSCT. To discover important relapse-associated mutations, we carried out longitudinal whole-exome sequencing analysis in matched diagnosis-remission-relapse post-HSCT samples from three adult patients with the most common subtype, Ph^−^ B-cell ALL (B-ALL). The mutations that we uncovered were followed up on by studying in an expanded Ph^−^ B-ALL cohort.

## RESULTS

### Mutations identified by whole-exome sequencing

We first performed whole-exome sequencing on germline DNA isolated from three relapsed Ph^−^ B-ALL cases with normal karyotype. Sequencing was performed at three distinct time points: at diagnosis (D), following complete remission (CR; after chemotherapy and before allo-HSCT), and at the time of relapse (TR; after allo-HSCT) (*i.e.*, discovery cohort; Supplementary Table 1). We then determined the mean exome coverage depth, which is defined as the mean number of reads that cover the captured coding sequence of a haploid reference. We found a 106-fold mean coverage depth, with 94.8% of the target exome covered by at least two reads and 89.4% covered by at least 10 reads (Supplementary Table 2).

Whole exome sequencing and bioinformatics analysis were carried out in a manner that is illustrated in the workflow chart in Supplementary Figure 1. We utilized an in-house software program to help identify candidate relapse-associated somatic mutations. We compared variants that were identified in the bone marrow exome dataset with dbSNP130 (downloaded from http://www.ncbi.nlm.nih.gov) as well as with data from the 1000 Genomes Project (downloaded from http://www.1000genomes.org). The variants that were identified in leukemic D or TR samples were compared with germline variants that were present in the CR sample from the same individual. We focused our research on the mutations that passed our quality control analyses and were also predicted to cause protein-coding changes. We also employed literature searches to identify genes thats altered structure and/or expression were associated with cancer and other human diseases. With these combined approaches, we successfully identified 102 potential somatic sequence alterations. These included 87 single-nucleotide variants (SNVs), 13 small insertions or deletions (indels) that inappropriately shifted the open reading frame, as well as two splice-site mutations. After validating D, CR and TR DNA samples, in addition to their respective donor samples by Sanger sequencing, 25 candidate relapse-associated somatic mutations were confirmed. These included 21 nonsynonymous substitutions and four indels in 23 unique genes, including the gene *USP54* that was commonly mutated in two patients (Supplementary Table 3). In addition to gene mutations that are known to be involved in leukemogenesis, such as ones in *MYC* and *KR*AS, we also identified mutations within *PTPN21*, *TBX21*, *USP54, USP11, NCOR2, CSPP1* that have never before been identified in human leukemia (Table 1).

**Table 1:** Candidate relapse-associated mutated genes identified in three primary tumor-relapse pairs by whole-exome sequencing

UPN	Primary tumor-specific mutated genes	Somatic mutations shared by the primary and relapsed tumor	Relapsed tumor-specific mutated genes
**ALL001**	None	*OXTR, TBX21, STEAP3, SLURP1, CSPP1, KDM6A, PTPN21*	None
**ALL002**	*NRF1, MARCKS, USP11, ELK1, MYC*	*CREBBP, RGS11*	*USP54, NCOR2*
**ALL003**	*USP54, GABRA3, KRAS, SETD2*	None	*MYH7, NYNRIN, ODZ1, ZIC3*

UPN, unique patient number

### Validation of relapse-associated mutations in an extended ALL cohort

To explore these findings further, we employed target gene sequencing in an extended validation cohort to identify mutations in entire coding-regions of each of the 23 identified genes. This cohort included 58 Ph^−^ adult B-ALL patients that had normal karyotypes (*i.e.*, extension cohort). Furthermore, taking into account the sample size limitation of whole-exome sequencing, we also decided to include nine additional genes in our analyses: *PAX5*, *CDKN2A/B*, *IKZF1/IKAROS*, *VPREB1*, *EBF1*, *TCF3*/*E2A*, *NR3C1* and *ETV6*. In recent studies using genome-wide copy number analyses, expression arrays, and methylation analyses, mutations within each of these genes were identified in relapsed ALL child patients following chemotherapy [10–17].

All 58 patients that participated in this study were subjected to T-cell replete allo-HSCT at our Bone Marrow Transplantation Center (Hangzhou), between March 2004 and April 2008. By August 2014, 28 patients had experienced relapse, with a median time of 7.5 months and a range from 2–33 months following allo-HSCT. Nonetheless, 30 patients did not experience any relapse after a median follow-up time of 50.75 months post-HSCT (with a range from 24–80 months). Each individual patient underwent a myeloablative conditioning regimen, consisting of 3.2 mg/kg/d IV of busulfan for four days and 60 mg/kg/d IV of cyclophosphamide for two days. This regimen also involved a graft-versus-host disease prophylaxis, which consisted of cyclosporin A (CSA) methotrexate, and low-dose mycophenolate mofetil. From day seven, CSA was given intravenously at 2.5 mg/kg/day, with a target blood level of 200–300 ng/mL. This dosage was tapered according to chimeric status and GVHD evidence during the second month following transplantation. On day one, MMF was delivered orally at 500 mg/day and withdrawn on day 100. In addition, MTX was given at 15 mg/m^2^ on day one, and then lowered to 10 mg/m^2^ on days three and six. There were no significant differences detected in age, cytogenetics, time from diagnosis to HSCT, disease status at the time of transplantation, or donor type between patients with or without relapse (Supplementary Table 4). The percent of patient versus donor cells in relapsed samples was quantitated using short tandem repeat analysis (Supplementary Table 5).

We performed targeted gene sequencing on samples that were collected at three distinct time points: at diagnosis, during CR, and at relapse post-HSCT for relapsed patients. Samples were obtained at both diagnosis and CR for non-relapsed patients. All respective donor DNA samples were also subjected to targeted gene sequencing for all genes of interest. Of the 28 relapsed cases, 13 patients (46.4 %) harbored somatic mutations, including 16 protein-coding variants within eight genes. It is important to note that if an individual had different mutations either in the same gene or in the different genes simutaneously, then it was counted only once. Nevertheless, out of the 30 non-relapsed cases, only five (16.7%) contained somatic mutations. Remarkably, relapsed ALL cases acquired somatic gene mutations significantly more frequently than non-relapsed cases (*p* = 0.023; two-sided Fisher's exact test).

**Figure 1:**
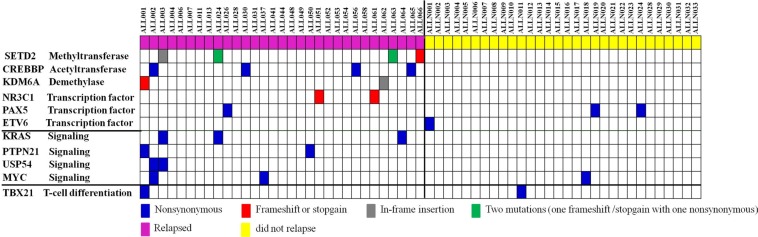
Whole-exome sequencing and target gene sequencing reveal recurrently mutated genes in Ph^−^ B-ALL patients Matched tumor-remission-relapse post-HSCT samples from 31 relapsed Ph^−^ B-ALL patients and matched tumor-remission samples from 30 non-relapsed Ph^−^ B-ALL patients were sequenced and somatic mutations detected in diagnosis, or gained at the time of relapse, or retained from diagnosis to relapse in every tumor sample were identified. Alteration type is identified for each mutation as frameshift/stopgain, nonsynonymous or in-frame insertion.

Sequence analysis of the 28 matched relapsed samples displayed a striking mutation enrichment in epigenetic regulators. Ten individual cases (35.7%) contained a mutation, which accounts for 76.9% (10/13) of the somatic mutations that were identified in relapsed patients. Out of the 10 patients, four of them gained an additional mutation in an epigenetic regulator at the time of relapse, whereas six other patients retained the same mutations from diagnosis to relapse. In total, 31 relapsed patients in the discovery (*n* = 3) and extension (*n* = 28) cohorts, showed recurring mutations in the following epigenetic regulators: *SETD2*, *CREBBP*, *KDM6A,* and two important transcription factors with epigenetic modulating functions (*NR3C1* and *PAX5)* (Figure 1, Table 2, Supplementary Figures 2 and 3). Notably, both *SETD2* and *CREBBP* were the most frequently mutated genes (12.9%). We also identified recurring mutations in the signaling factors *KRAS, PTPN21, MYC and USP54* from relapsed samples (Figure 1, Table 2, Supplementary Figure 4). In our study, relapse associated mutations mean that gene mutations according with these two conditions, first, the mutations are either retained in tumors from diagnosis until relapse or that are selectively acquired at relapse post-HSCT. Second, they were mutated in at least two relapsed cases but were not mutated in any of the non-relapsed cases. In the ten recurring mutated genes, seven genes (SETD2, *CREBBP*, *KDM6A*, *NR3C1, KRAS, PTPN21* and *USP54)* were mutated in at least two relapsed cases but were not mutated in any of the non-relapsed cases. This therefore suggests that these discovered mutations are relapse-associated and involved in pathogenesis of Ph^−^ adult ALL relapse post-HSCT. Additional three genes (*MYC*, *TBX21*, and *PAX5*) were selectively mutated in leukemic samples irrespective of whether relapse had occurred, but not in remission or healthy donor samples, implying that these mutations are involved in the initiating events of adult ALL (Figure 1, Table 2).

**Table 2:** Recurring gene mutations in the discovery and extension ALL cohorts

Gene	Positions	Mutation type	Allele change	Amino acid change	Relapsed cases		Non-relapsed cases (UPN) in diagnosis
					UPN^a^	Mutation distribution	
*CREBBP*	Chr 16:NM_004380	Nonsynonymous	Exon26: c.4337G > A	p.Arg1446 His	ALL002	Diagnosis-relapse shared	—
		Nonsynonymous	Exon30: c.5098C > A	p.Q1700K	ALL030	Relapse-specific	—
		Nonsynonymous	Exon20: c.3710G > A	p.C1237Y	ALL056	Diagnosis-relapse shared	—
		Nonsynonymous	Exon27: c.4463C > T	p.P1488L	ALL065	Diagnosis-relapse shared	—
*KRAS*	Chr 12: NM_004985	Nonsynonymous	Exon2:c.34G > C	p.G12R	ALL003	Diagnosis-specific	—
		Nonsynonymous	Exon3:c.173C > T	p.T58I	ALL064	Diagnosis-relapse shared	—
		Nonsynonymous	Exon2:c.38G > A	p.G13D	ALL024	Diagnosis-relapse shared	—
*PTPN21*	Chr 14: NM_007039	Nonsynonymous	Exon13: c.1573C > G	p. Pro 525 Ala	ALL001	Diagnosis-relapse shared	—
		Nonsynonymous	Exon13: c.1975G > A	p. Ala 659 Thr	ALL001	Diagnosis-relapse shared	—
		Nonsynonymous	Exon13: c.1514C > A	p.P505Q	ALL050	Relapse-specif ic	—
*KDM6A*	Chr X:NM_021140	Frameshift	Exon17: c.2563_2564 insG	p.Asn855Argfs × 20	ALL001	Diagnosis-relapse shared	—
		In-frame	Exon28:c.4031_4051del insGGG	p.Val1344_Arg1351delinsGlyGly	ALL062	Diagnosis-relapse shared	—
*USP54*	Chr 10: NM_152586	Nonsynonymous	Exon18: c.3130A > T	p. Thr 1044 Ser	ALL002	Relapse-specific	—
		Nonsynonymous	Exon20: c.4250G > A	p. Arg 1417 His	ALL003	Diagnosis-specific	—
*NR3C1*	Chr 5: NM_001204260	Frameshift	Exon2:c.431_431delinsAG	p.D144Efs×11	ALL051	Relapse-specific	—
		Stopgain^c^	Exon2:c.640C > T	p.Q214X	ALL061	Relapse-specific	—
*MYC*	Chr 8: NM_002467	Nonsynonymous	Exon2: c.221C > G	p. Pro 74 Arg	ALL002	Diagnosis-specific	—
		Nonsynonymous	Exon2: c.293G > A	p.R98Q	ALL037	Relapse-specific	—
		Nonsynonymous	Exon2: c.223C > T	p.P75S	—	—	ALLN018
*TBX21*	Chr 17: NM_013351	Frameshift	Exon4: c.875_876 insGG	p.Phe292Leufs×12	ALL001	Diagnosis-relapse shared	—
		Nonsynonymous	Exon1: c.76G > C	p.A26P	—	—	ALLN011
*SETD2*	Chr3:NM_014159	In-frame	Exon20: c.7517_7518 insGGT	p.Lys2506_His2507insVal	ALL003	Diagnosis-specific	—
		Stopgain^c^	Exon11: c.5345G > A	p.W1782X	ALL024	Diagnosis-relapse shared	—
		Nonsynonymous	Exon6: c.4808A > G	p.H1603R	ALL024	Diagnosis-relapse shared	—
		Nonsynonymous	Exon7: c.4874G > A	p.R1625H	ALL063	Diagnosis-relapse shared	—
		Frameshift	Exon3: c.1508_1509insTTCG	p.Glu503Aspfs × 18	ALL063	Diagnosis-relapse shared	—
		Frameshift	Exon17: c.7199_7201delinsCT	p.Asp2400Alafs × 11	ALL066	Relapse-specific	—
*PAX5*	Chr9:NM_016734	Nonsynonymous	Exon2:c.77T > G	p.V26G	—	—	ALLN019
		Nonsynonymous	Exon2:c.191G > T	p.C64F	—	—	ALLN024
		Nonsynonymous	Exon3:c.239C > G	p.P80R	ALL026	Diagnosis-relapse shared	—

a UPN, unique patient number.

^b^A total of 31 relapse patients were included in the discovery (*n* = 3) and extension (*n* = 28) ALL cohorts.

c Stopgain is defined as a point mutation within a DNA sequence that results in either a premature stop codon or a nonsense codon at the mutated site.

**Figure 2:**
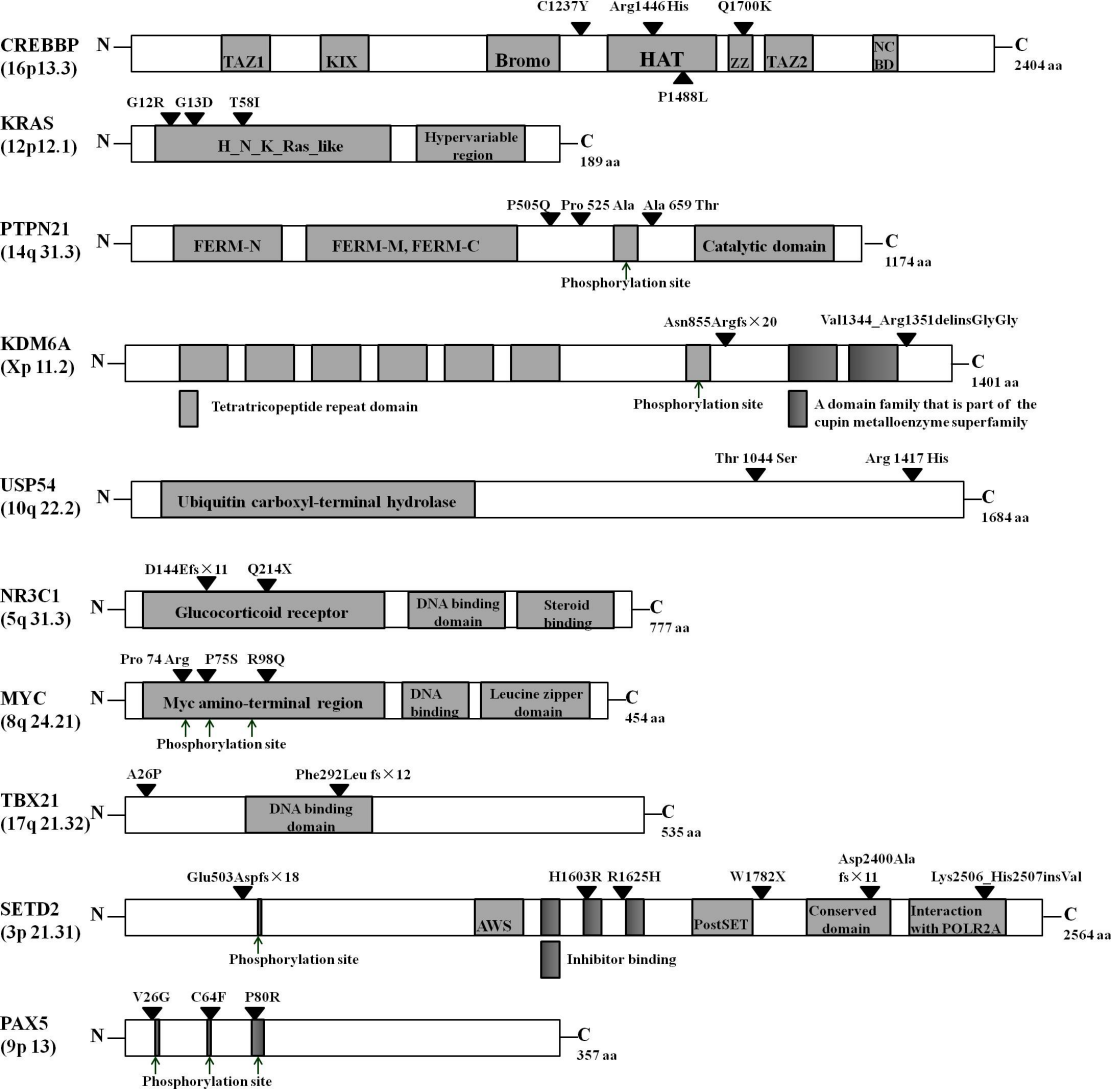
Distribution of mutated gene alterations The alterations encoded by confirmed somatic mutations are indicated by black arrowheads. TAZ1, transcriptional-adaptor zinc-finger 1; KIX, KID-binding domain; Bromo, bromodomain; HAT, histone acetyltransferase domain; ZZ, zinc-binding domain near the dystrophin WW domain; NCBD, nuclear-receptor coactivator-binding domain; FERM-N, FERM N-terminal domain; FERM-M, FERM central domain; FERM-C, FERM C-terminal domain; AWS, associated with SET domains.

Mutations within the ten recurrently mutated genes exclusively occurred at sites that are highly conserved across species (Supplementary Figures 5 and 6). Furthermore, mutations were found in either major functional domains or proximal to phosphorylation sites, which is likely to perturb proper protein function (Figure 2). Survival data analyses from the study cohort revealed significant differences between patients with relapse and those who showed no signs of relapse. With the exception of one patient, all other relapsed patients died.

### Patterns of clonal evolution from diagnosis to relapse in ALL

**Figure 3:**
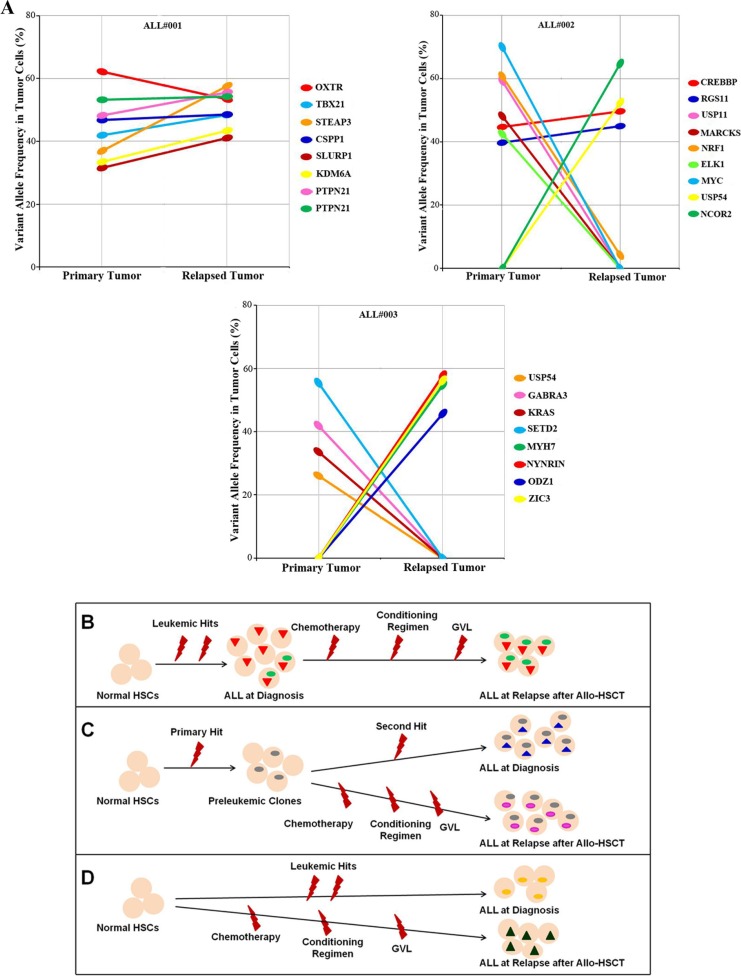
Graph of clonal evolution patterns from primary and relapsed tumors after Allo-HSCT (**A**) Frequencies of validated somatic mutant alleles identified by whole-exome sequencing in diagnosed and relapsed tumors from three initial ALL patients. The relationships between mutations in the primary tumor and relapsed tumor are indicated by the lines linking them together. (**B**) The relapse clone arose from a subclone that was already existent within the diagnosed tumor. (**C**) The relapse clone originated from a common progenitor to the diagnosis clone, but had acquired new mutations while retaining some but not all of those found in the original tumor. (**D**) Leukemia recurrence following allo-HSCT that has a completely distinct set of mutations from the primary tumor, which differentiates it as a second malignancy. Dots and trilateral with different colors represent distinct mutations.

The whole-exome sequencing dataset of the three relapsed cases provided us with the ability to accurately quantitate mutant allele frequencies for each of the validated somatic SNVs in every diagnosed and relapsed tumors. After adjusting for clonal tumor cell population size in each ALL sample, sequence variant fluctuation from ALL progression to relapse suggested that there are heterogeneous clonal evolution patterns within individual patients (Table 3). For example, the primary and relapsed tumors in patient ALL001 had eight concordant somatic mutations in seven distinct genes. Importantly, no one mutation was exclusive to either tumor (Table 1). Furthermore, the variant frequencies in the tumor of five mutations within the *OXTR*, *TBX21*, *CSPP1*, and *PTPN21* genes ranged from 40%–50% in the primary tumor, thus suggesting the likelihood that these were present in virtually all tumor cells at the onset (heterozygosity). Therefore, the subclones were derived from the tumor clone that harbored all five mutations. It is important to note that a particular subclone with the set of gene mutations (*e.g. STEAP3*, *SLURP1* and *KDM6A*) within the primary tumor may have grown out or outcompeted the others and survived both chemotherapy selection and allo-HSCT treatment to evolve into the dominant clone at relapse. This scenario is likely, since the variant frequencies in the tumor of these genes were ∼30% within the primary tumor and were increased significantly to 40%–50% upon relapse (Figure 3A).

**Table 3:** Readcount data for 28 somatic mutations in three initial whole-exome sequencing patients

Case UPN	Gene	Amino acid change	Solexa primary tumor reads				Solexa relapsed tumor reads				Solexa CR sample reads		
			% Blasts	Variant	WT	%Variant in tumor	% Blasts	Variant	WT	%Variant in tumor	Variant	WT	%Variant in tumor
ALL001	*OXTR*	p.Ser377Ile	90	23	18	62.3	92	32	33	53.5	0	83	0
ALL001	*TBX21*	p.Phe292LeufsX12	90	25	41	42.1	92	46	57	48.5	0	114	0
ALL001	*STEAP3*	p. Pro 210 Leu	90	5	10	37	92	17	15	57.7	0	73	0
ALL001	*SLURP1*	p. Cys 25 Phe	90	10	25	31.7	92	22	36	41.2	0	118	0
ALL001	*CSPP1*	p. Leu 1103 Pro	90	30	41	46.9	92	21	26	48.6	0	97	0
ALL001	*KDM6A*	p.Asn855ArgfsX20	90	35	81	33.5	92	44	66	43.5	0	70	0
ALL001	*PTPN21*	p. Pro 525 Ala	90	10	13	48.3	92	21	20	55.7	0	107	0
		p. Ala 659 Thr	90	12	13	53.3	92	20	21	53.0	0	29	0
ALL002	*CREBBP*	p. Arg 1446 His	95	37	50	44.8	33	21	107	49.7	0	81	0
ALL002	*RGS11*	p. Arg409Arg	95	14	23	39.8	33	21	120	45.1	0	23	0
ALL002	*USP54*	p. Arg 1417 His	95	0	50	0	33	13	62	52.5	0	55	0
ALL002	*NCOR2*	p. Ala 2013 Thr	95	0	37	0	33	7	26	64.8	0	22	0
ALL002	*NRF1*	p. Lys 226 Ile	95	44	33	60.9	33	1	72	4.2	0	42	0
ALL002	*MARCKS*	p. Ala15Thr	95	11	13	48.2	33	0	49	0	0	23	0
ALL002	*USP11*	p. Ala739Thr	95	31	24	59.3	33	0	44	0	0	40	0
ALL002	*ELK1*	p. Pro 238 Leu	95	21	31	42.5	33	0	47	0	0	42	0
ALL002	*MYC*	p. Pro74Arg	95	10	5	70.2	33	0	37	0	0	31	0
ALL003	*USP54*	p. Thr 1044 Ser	93	37	115	26.2	74	0	118	0	6	166	3.5
ALL003	*GABRA3*	p. Ser60Gly	93	25	39	42	74	0	39	0	0	70	0
ALL003	*KRAS*	p. Gly12Arg	93	39	85	33.8	74	0	138	0	0	107	0
ALL003	*SETD2*	p.Lys2506_His2507insVal	93	61	57	55.6	74	0	83	0	6	107	5.3
ALL003	*MYH7*	p. Leu1104 Gln	93	0	73	0	74	26	38	54.9	0	65	0
ALL003	*NYNRIN*	p.Thr652SerfsX50	93	0	24	0	74	33	44	57.9	0	37	0
ALL003	*ODZ1*	—	93	0	54	0	74	19	37	45.8	0	60	0
ALL003	*ZIC3*	p. Arg123His	93	0	39	0	74	10	14	56.3	0	34	0

Clonal evolution in patient ALL002 was characterized by a divergence in mutational profile from the original tumor. While all of the tumor cells at both diagnosis and relapse shared the two SNVs in *CREBBP* and *RGS11*, five additional mutations in *NRF1*, *MARCKS*, *USP11*, *ELK1*, and *MYC* were detected at diagnosis. Furthermore, the relapsed tumor acquired two relapse-specific mutations in *USP54* and *NCOR2* (Figure 3A). These data suggest that while tumor cell clones from both diagnosis and relapse originated from a common progenitor, these progenitors ultimately acquired distinct additional mutations that all promoted leukemia relapse.

Additionally, patient ALL003 revealed a distinctive clonal evolution pattern where uncommon mutations were observed in both initial and relapsed leukemia. At the time of diagnosis, the tumor cells had four distinct SNVs in *USP54*, *GABRA3*, *KRAS* and *SETD2*, while the relapsed tumor acquired four additional unique mutations in *MYH7*, *NYNRIN*, *ODZ1* and *ZIC3* (Figure 3A).

## DISCUSSION

Gene mutations that are either retained in tumors from diagnosis until relapse or that are selectively acquired at relapse post-HSCT offer great insight into processes that intensively alter the leukemic cell fitness (*e.g.,* proliferation rate and/or survival). While functional evaluation of the revealed mutations require extensive further exploration, our study provides the first evidence that somatic mutations in both epigenetic regulators and signaling factors are involved in leukemia relapse pathogenesis following allo-HSCT in Ph^−^ adult B-ALL. These recurrently mutated genes included *SETD2*, *CREBBP*, *KDM6A*, *NR3C1, KRAS, PTPN21,* and *USP54*, with the most frequent mutations detected in the epigenetic regulators *SETD2* and *CREBBP* of relapsed patients. *SETD2* is the only mammalian histone H3K36 trimethyltransferase that has been suggested to display tumor suppressor activity in breast cancer and renal cell carcinoma [18–20]. Inactivating lesions in *SETD2* have been recently implicated in 22.2% of MLL gene-rearranged leukemia pathogenesis and also in 4.6% of patients with leukemia that did not have *MLL* rearrangements [21]. Armstrong SA *et al.* also indentified that mutations in SETD2 are gained during relapse in pediatric ALL after chemotherapy [22]. Our data are the first to provide insight into the association between *SETD2* mutations and a risk of relapse post-HSCT in Ph^−^ adult B-ALL. The CREB-binding protein (*CREBBP*) is a large, multifunctional protein that facilitates transcriptional coactivation and acetylation of histone and non-histone targets [16]. Our study adds additional insight to previous findings that identified *CREBBP* mutations in 18.3% of relapsed childhood ALL cases following chemotherapy. These mutations were shown to promote the dysregulation of glucocorticoid-responsive genes [23]. Interestingly from our study, somatic mutations in *NR3C1* were selectively acquired at relapse from two distinct cases. *NR3C1* encodes a transcription factor/glucocorticoid receptor, which modulates gene expression via binding to the promoters of glucocorticoid responsive genes to activate their expression, or by protein-protein interactions with other transcription factors. Loss-of-function mutations in *NR3C1* has been associated with both chemotherapy and GVL tolerance. This topic requires further investigation in order to define the role of *NR3C1* in the prognosis and treatment failure of adult ALL. Another epigenetic regulator, the lysine-specific demethylase 6A encoded by the *KDM6A* gene*,* catalyzes the demethylation of histone H3 [24] and regulates both stem cell migration and hematopoiesis [25, 26].

We also found a significant mutational enrichment within the tumor-associated transmembrane signal transduction genes *KRAS*, *PTPN21*, and *USP54* of relapsed patients. *KRAS* is an oncogene in the Ras-MAPK signaling pathway, and mutations within this gene have been associated with leukemogenesis and hematologic malignancies [27–29]. The protein tyrosine phosphatase non-receptor type 21 gene (*PTPN21*) encodes a member of the protein tyrosine phosphatase (PTP) family, which is involved in PI3K-AKT, MAPK, and JAK-STAT signaling. Additionally, mutations in *PTPN21* have been reported in both bladder cancer [30] and colorectal tumors [31]. *USP54* is a ubiquitin-specific peptidase that activates the TNFα-NF-κB pathway, and the upregulation of this gene family has been linked to both lung [32] and pancreatic [33] cancers, as well as to Wilms' tumors [34]. This study is the first to confirm that both *PTPN21* and *USP54* are mutated in human leukemia.

Furthermore, we examined the clonal evolution of leukemic cells from diagnosis to relapse following allo-HSCT, which extends the findings of previous studies on clonal evolution in leukemia relapse after chemotherapy [12, 35, 36]. Our data suggest that the cells that are present in relapsed specimens may be genetically related to the diagnosed tumor, and could either arise from an existing subclone that was not thoroughly eradicated by allo-HSCT therapy (Figure 3B), or originate from the same progenitor but acquire additional mutations to those found in the original tumor (Figure 3C). In contrast, some cases of leukemia recurrence following allo-HSCT may result from a secondary malignancy that exhibits a distinct set of mutations (Figure 3D). There are clonal heterogeneity in the primary tumor followed by dynamic clonal evolution at relapse, including the addition of new mutations that may be relevant for relapse pathogenesis, which is a common feature shared in leukemia relapse after chemotherapy and after allo-HSCT. Although pre-HSCT chemotherapy and allo-HSCT are required for the treatment of patients with hematologic malignancies, our data raise the possibility that this treatment combination promotes relapse by inducing genetic instability and chromosomal damage. In addition, these treatments could dysregulate homeostasis, cause sustained aberrant antigenic stimulation, or promote impaired immune surveillance.

While the findings reported here are limited by the number of patients analyzed and would greatly benefit from further investigation, we are the first to identify genetic causes of leukemia relapse following allo-HSCT based on the largest dataset ever collected from adult patients with Ph^−^ B-ALL. The findings revealed in this study have several promising clinical implications. Firstly, the heterogeneous clonal evolution patterns that underlie ALL progression from diagnosis to relapse following allo-HSCT can inform therapeutic decisions for relapsed patients and help prevent the use of ineffective treatments. Secondly, this study suggests that epigenetic modifiers provide novel and attractive targets for therapeutic intervention in ALL relapse post-HSCT. The initial incorporation of epigenetic therapies in either pre-HSCT or prophylaxis therapy post-HSCT, particularly in patients with mutations identified in *de novo* ALL, could be a potent strategy for preventing relapse following allo-HSCT.

## MATERIALS AND METHODS

### Patients and sample collection

From March 2004 to April 2008, 61 individual Ph^−^ adult B-ALL patients with normal karyotype, who underwent T-cell replete allo-HSCT at our Bone Marrow Transplantation Center (Hangzhou), were included in the present genomic analysis. Genomic DNA samples were obtained from specimens collected at varying stages: during the time of disease onset, following complete remission (CR) (minimal residual disease level < 1 × 10^−4^) after chemotherapy (but before HSCT), and at the time of relapse. Mononuclear-cell-enriched bone marrow samples from both relapsed patients and stem cell donors were taken for the relapsed category. Diagnostic samples and relapsed samples had > 30% blasts. The protocol was approved by the ethics review committee of the First Affiliated Hospital of Zhejiang University School of Medicine, and all samples were obtained according to the guidelines of the local ethical committees.

### Measurements of cells from relapsed patient samples

After donor hematopoietic stem cells were successfully engrafted into the patient, it was possible that some of the patients were in a donor-patient chimerism status when they experienced relapse post-HSCT, which was of some concern. This suggests that the relapsed sample could possibly represent a mixture of both the patient's and his/her respective donor's cells. We genotyped 15 highly polymorphic short tandem repeat (STR) loci using the AmpFLSTR^®^ Identifiler^®^ PCR Amplification Kit (Applied Biosystems, Waltham, MA, USA; Life Technologies, Carlsbad, CA, USA) using the samples that were obtained at CR pre-HSCT and relapsed post-HSCT from the same patients, as well as his/her respective donor's blood sample. These 15 STR loci were characterized as “informative” if at least one CR-specific allele and one donor-specific allele were identified in the relapsed sample. In addition, these two alleles had to have less than two repeat units of difference in size. The ratio of the peak height of the CR-specific alleles to the sum of both the CR- and donor-specific alleles were calculated for each informative STR locus, and the percentage of each patient's cells in relapsed samples was estimated using the average ratio values for all “informative” STR loci.

### Whole-exome sequencing

Whole-exome sequencing was conducted for nine genomic DNA samples from three relapsed cases with Ph^−^ B-ALL at three specific time points: diagnosis (−D), CR during chemotherapy but before allo-HSCT (−CR), and relapse following allo-HSCT (−TR) (discovery cohort, Supplementary Table 1). Approximately 4 μg of genomic DNA of each sample was fragmentized into 100-800 bp pieces with a peak size of ∼250 bp using NEBNext^®^ dsDNA Fragmentase^®^ (New England Biolabs, Ipswich, MA, USA), followed by end-repairing, dA-Tailing and adaptor ligation using the NEBNext^®^ DNA Library Prep Reagent Set from Illumina^®^ (New England Biolabs, Ipswich, MA, USA). The adaptor-ligated DNA fragments were fractionated by 2% agarose gel electrophoresis and fragments of the desired size (300–400 bp) were excised. The extracted DNA was amplified in 10 PCR cycles using PE primers (Illumina, San Diego, CA, USA) and Phusion DNA polymerase (New England Biolabs, Ipswich, MA, USA). The PCR products were then subjected to exome sequence capture using the Illumina Truseq Exome Enrichment kit V3, which contains a 31.3 Mb CCDS (97.2% of the US National Center for Biotechnology Information CCDS Database) region across ∼20,794 genes within 62 Mb of coding exons, according to the manufacturer's manual (Illumina, San Diego, CA, USA). The enriched elution was amplified in 10 PCR cycles using PE primers and Phusion DNA polymerase. The amplicons were size-checked and quantitated using a BioAnalyzer 2100, and then subjected to 2 × 100 bp paired-end massively parallel sequencing using a Genome Anlayzer IIx platform (Illumina, San Diego, CA, USA)

### Massively parallel sequencing data processing and SNV/indel calling

Before variant calling, sequence alignment files were generated to duplicate removal, local realignment around known indels and base quality recalibration using the Genome Analysis Toolkit (GATK). Variations that included single-nucleotide variants (SNVs) and small insertions or deletions (indels) were identified using both the VarScan 2.2.7 software package (http://www.ncbi.nlm.nih.gov/pubmed/22300766) as well as the variant quality score recalibration (VQSR) protocol in GATK, and further filtered using a recommended threshold value (mapping quality > 30, base quality >15, and read numbers > 3). Then, SNVs available at dbSNP130 (hg19) as well as those reported by the 1000 Genomes Project were filtered out from the output files using the ANNOVAR (http://nar.oxfordjournals.org/content/38/16/e164). Variant calling was performed separately for each individual sample.

### Candidate somatic mutation selection and sanger sequencing validation

We identified putative somatic mutations by comparing each individual tumor (either diagnostic samples or relapsed samples) to normal (CR samples) from the same patient. SNVs/indels identified in the samples obtained at disease onset and/or relapse but not at CR were considered as candidate somatic mutations. These somatic SNVs/indels with a high enough confidence level (with ≥ 15% allele frequency, 20x coverage in either tumor sample and < 0.5% in the remission sample) were selected as candidate somatic mutations. Sanger sequencing was applied in order to validate the candidate variants in matched samples that were obtained at diagnosis, CR and relapse from each individual using the BigDye^®^ Terminator v3.1 Cycle Sequencing Kit (Applied Biosystems, Waltham, MA, USA). All of the respective donor DNA samples also received targeted re-evaluation using Sanger sequencing validation to rule out those SNVs/indels that were selectively identified in relapsed samples but actually originated from donor DNA.

### Targeted next-generation sequencing

To screen relapse-associated gene mutations and define the frequencies of gene mutations identified by whole-exome sequencing analysis, we carried out further whole coding-region sequencing for target genes in an extended validation cohort. Genomic DNA from samples at diagnosis, CR, relapse and from donors in the extended cohort were amplified using Multiplex-PCR reactions to capture targeted genes, which were then subjected to DNA sequencing on the Illumina platform as described above. All candidate somatic SNV/indels that were identified by whole coding-region sequencing for targeted genes were also validated by Sanger sequencing using nonamplified DNA specimens from patients at diagnosis, CR, and relapse, as well as from donors.

### Statistical analysis

Clinical features between relapsed patients and non-relapsed patients were compared by using Independent-Samples *T* tests and Fisher's exact tests. Statistical analyses were performed using SPSS software version 16.0. All probability values were generated from two-sided tests. *P* < 0.05 was considered as statistically significant, and *p* values spanning between 0.05 and 0.1 were characterized as representing a trend.

Supplementary Information accompanies this paper on the Oncotarget website (http://www.impactjournals.com/oncotarget).

## SUPPLEMENTARY MATERIALS TABLES AND FIGURES


