# Supplementary material for: Mutations in epigenetic regulators are involved in acute lymphoblastic leukemia relapse following allogeneic hematopoietic stem cell transplantation
Source: Oncotarget. 2015 Oct 30;7(3):2696–708. doi: 10.18632/oncotarget.6259 (PMC4823065; doi:10.18632/oncotarget.6259)
Supplement: Supplementary file 1 [file oncotarget-07-2696-s001.pdf]

Mutations in epigenetic regulators are involved in acute lymphoblastic leukemia relapse following allogeneic hematopoietic cell transplantation

Supplementary Materials

Supplementary Table S1: Clinical characteristics of relapsed ALL patients in initial whole-exome sequencing set

Unique patient number (UPN)	Sex	Age	% BM leukemic cells at diagnosis sample	The number of courses of chemotherapy to achieve CR	The number of courses of chemotherapy before HSCT	Disease status at HSCT	Donor type	Months of relapse after HSCT	% BM leukemic cells at relapse sample	STR analysis of relapse sample
ALL001	Female	16	90	1	6	CR	Haploidentical related donor	6.3	92%	100% cells originated from patient
ALL002	Female	20	95	1	6	CR	Unrelated donor	33	33%	A donor-patient chimerism status with ~25% cells originated from patient
ALL003	Female	21	93	1	5	CR	HLA-matched sibling donor	28	74%	A donor-patient chimerism status with ~70% cells originated from patient

Supplementary Table S2: Sequencing depth and coverage of the nine matched whole-exome sequencing samples

Sample	On Bait Bases (M)	Mean_Target_Coverage	Covered ≥ 2x	Covered ≥ 10x	Covered ≥ 20x
ALL001-D	6407.503	102.8718	95.20%	91.82%	88.36%
ALL001-CR	7116.558	114.2556	92.51%	82.54%	75.08%
ALL001-TR	6436.359	103.335	95.58%	92.77%	89.94%
ALL002-D	6190.747	99.39178	94.02%	85.07%	74.47%
ALL002-CR	5717.431	91.79274	95.05%	88.57%	82.11%
ALL002-TR	6317.773	101.43	95.72%	93.01%	90.02%
ALL003-D	7471.011	119.9463	94.29%	87.87%	81.51%
ALL003-CR	7534.715	120.969	95.31%	89.85%	84.83%
ALL003-TR	6233.7	100.0814	95.84%	93.20%	90.07%

D: Diagnosis; CR: Complete remission following chemotherapy but before allo-HSCT; TR: Relapse after allo-HSCT.

Supplementary Table S3: Candidate relapse-associated somatic mutations with predicted functional alterations identified in 3 initial whole-exome sequencing patients

Gene	Mutation Type	Positions	Allele Change	Amino Acid Change	Case UPN	Sample Mutated
OXTR	Nonsynonymous	Chr 3: NM_000916: exon4: c.1130	C > A	p.Ser377Ile	ALL001	Diagnosis-relapse shared
TBX21	Frameshift	Chr17: NM_013351:exon4: c.875_876	insGG	p.Phe292LeufsX12	ALL001	Diagnosis-relapse shared
STEAP3	Nonsynonymous	Chr 2: NM_001008410:exon3: c. 629	C > T	p. Pro 210Leu	ALL001	Diagnosis-relapse shared
SLURP1	Nonsynonymous	Chr 8: NM_020427:exon2: c.74	G > T	p. Cys 25Phe	ALL001	Diagnosis-relapse shared
CSPP1	Nonsynonymous	Chr 8: NM_024790:exon27: c.3308	T > C	p. Leu 1103Pro	ALL001	Diagnosis-relapse shared
KDM6A	Frameshift	Chr X: NM_021140:exon17: c.2563_2564	insG	p.Asn855ArgfsX20	ALL001	Diagnosis-relapse shared
PTPN21	Nonsynonymous	Chr 14: NM_007039:exon13: c.1573	C > G	p. Pro 525Ala	ALL001	Diagnosis-relapse shared
	Nonsynonymous	Chr 14: NM_007039:exon13: c.1975	G > A	p. Ala 659Thr	ALL001	Diagnosis-relapse shared
CREBBP	Nonsynonymous	Chr 16: NM_004380:exon26:c.4337	G > A	p. Arg 1446His	ALL002	Diagnosis-relapse shared
RGS11	Splicing	Chr 16: NM_003834:exon17:c.1227	C > T	p. Arg409Arg	ALL002	Diagnosis-relapse shared
USP54	Nonsynonymous	Chr 10: NM_152586: exon20: c.4250	G > A	p. Arg 1417His	ALL002	Relapse-specific
	Nonsynonymous	Chr 10: NM_152586: exon18: c.3130	A > T	p. Thr 1044Ser	ALL003	Diagnosis-specific
NCOR2	Nonsynonymous	Chr 12: NM_006312:exon40:c.6037	G > A	p. Ala 2013Thr	ALL002	Relapse-specific
NRF1	Nonsynonymous	Chr 16: NM_005011:exon6:c.677	A > T	p. Lys 226Ile	ALL002	Diagnosis-specific
MARCKS	Nonsynonymous	Chr 6: NM_002356:exon1:c.43	G > A	p. Ala15Thr	ALL002	Diagnosis-specific
USP11	Nonsynonymous	Chr X : NM_004651:exon15:c.2215	G > A	p. Ala739Thr	ALL002	Diagnosis-specific
ELK1	Nonsynonymous	Chr X: NM_005229:exon4:c.713	C > T	p. Pro 238Leu	ALL002	Diagnosis-specific
MYC	Nonsynonymous	Chr 8: NM_002467:exon2:c.221	C > G	p. Pro74Arg	ALL002	Diagnosis-specific
GABRA3	Nonsynonymous	Chr X: NM_000808:exon3:c.178	A > G	p. Ser60Gly	ALL003	Diagnosis-specific
KRAS	Nonsynonymous	Chr 12: NM_004985:exon2:c.34	G > C	p. Gly12Arg	ALL003	Diagnosis-specific
SETD2	In-frame	Chr3: NM_014159: exon20:c.7517_7518	insGGT	p.Lys2506_ His2507insVal	ALL003	Diagnosis-specific
MYH7	Nonsynonymous	Chr 14: NM_000257: exon26:c.3311	T > A	p. Leu1104Gln	ALL003	Relapse-specific

NYNRIN	Frameshift	Chr 14: NM_025081:exon4:c.1955_1956	delCA	p.Thr652Serfs X50	ALL003	Relapse-specific
ODZ1	Splicing	Chr X: NM_001163279:exon4:c.535 + 1	G > C	—	ALL003	Relapse-specific
ZIC3	Nonsynonymous	Chr X:NM_003413:exon1:c.368:	G > A	p. Arg123His	ALL003	Relapse-specific

Supplementary Table S4: Clinical characteristics of ALL patients in the extension cohort

Characteristics	Relapsed ALL (*n* = 28)	Non-relapsed ALL (*n* = 30)	*P*-value
Age (median, range), Years	25.5 (16–45)	28 (16–44)	0.198
Sex, *n* (%)			0.798
Male	16 (57.1)	16 (53.3)	
Female	12 (42.9)	14 (46.7)	
BM leukemia cells at diagnosis, (%)	87.5 (50–96.5)	90 (50–96.5)	0.928
The number of courses of chemotherapy to achieve CR, *n* (%)			0.473
1 Course	25 (89.3)	24 (80.0)	
≥ 2 Course	3 (10.7)	6 (20.0)	
The Number of courses of chemotherapy before HSCT, (median, range)	6 (4–15)	6 (3–20)	0.698
Time from diagnosis to HSCT, (median, range), Months	8 (5–17)	7.75 (3–24)	0.562
Disease status before HSCT, *n* (%)			0.726
CR1	23 (82.1)	26 (86.7)	
≥ CR2	5 (17.9)	4 (13.3)	
Donor type, *n* (%)			0.713
HLA-identical sibling	10 (35.7)	9 (30.0)	
Unrelated donor	13 (46.4)	13 (43.3)	
Haploidentical donor	5 (17.9)	8 (26.7)	
Donor-patient gender, *n* (%)			0.418
Male-male	9 (32.1)	5 (16.7)	
Male-female	5 (17.9)	8 (26.7)	
Female-male	6 (21.4)	10 (33.3)	
Female-female	8 (28.6)	7 (23.3)	

Supplementary Table S5: The percentage of patients' and donors' cells in relapsed samples from the extended cohort by STR analysis

UPN	Relapsed sample	
	Cells originated from patient (%)	Cells originated from donor (%)
**ALL004**	100	0
**ALL006**	61	39
**ALL007**	65	35
**ALL011**	100	0
**ALL013**	100	0
**ALL024**	100	0
**ALL026**	100	0
**ALL028**	100	0
**ALL030**	59	41
**ALL031**	100	0
**ALL037**	100	0
**ALL041**	100	0
**ALL044**	66	34
**ALL048**	77	23
**ALL049**	61	39
**ALL050**	77	23
**ALL051**	100	0
**ALL052**	66	34
**ALL053**	100	0
**ALL054**	47	53
**ALL056**	100	0
**ALL058**	36	64
**ALL061**	100	0
**ALL062**	100	0
**ALL063**	100	0
**ALL064**	100	0
**ALL065**	100	0
**ALL066**	100	0

UPN denotes unique patient number

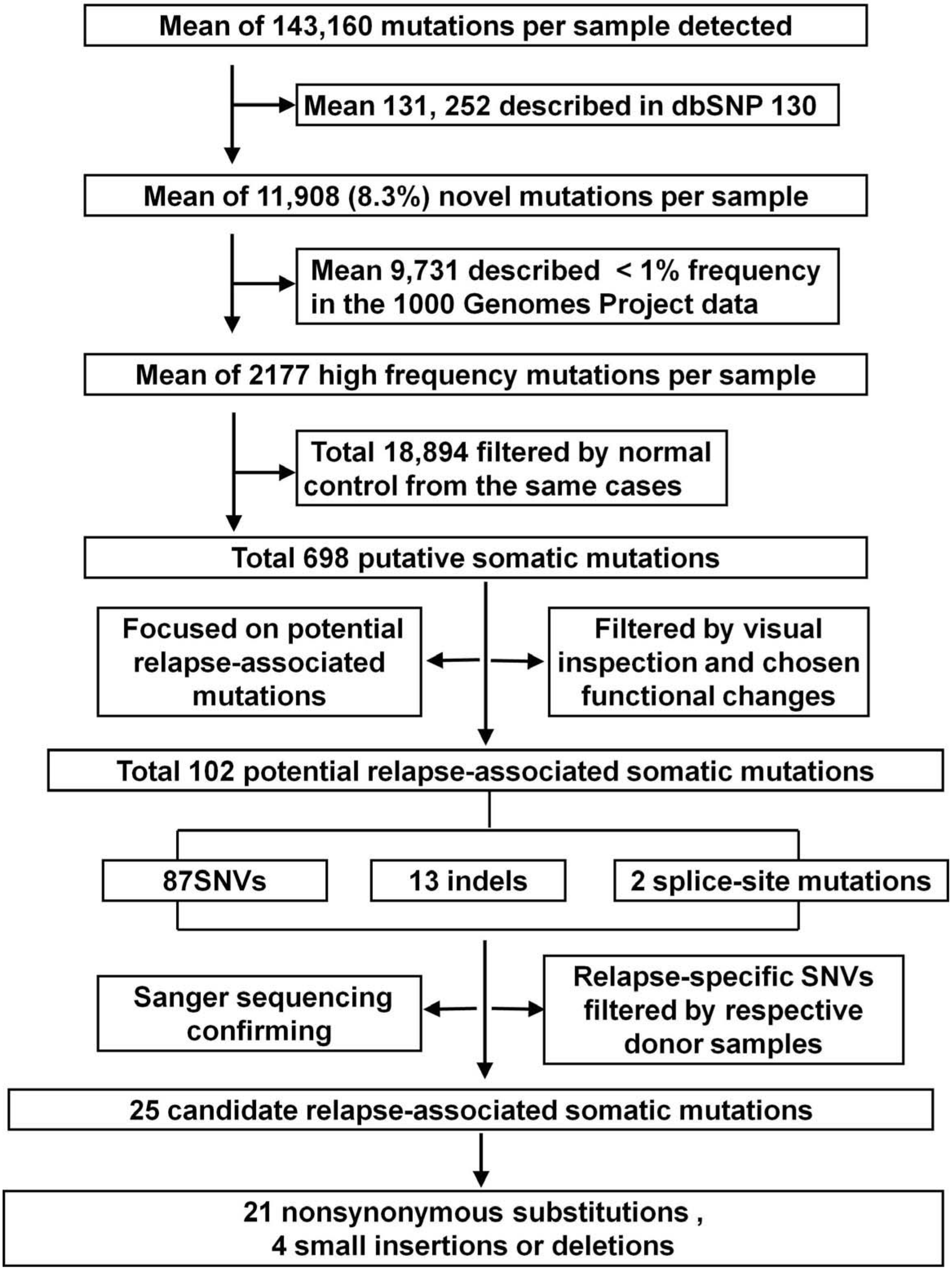

Supplementary Figure S1: Flow chart for identification of candidate relapse-associated somatic mutations from database of 3 initial whole-exome sequencing patients.

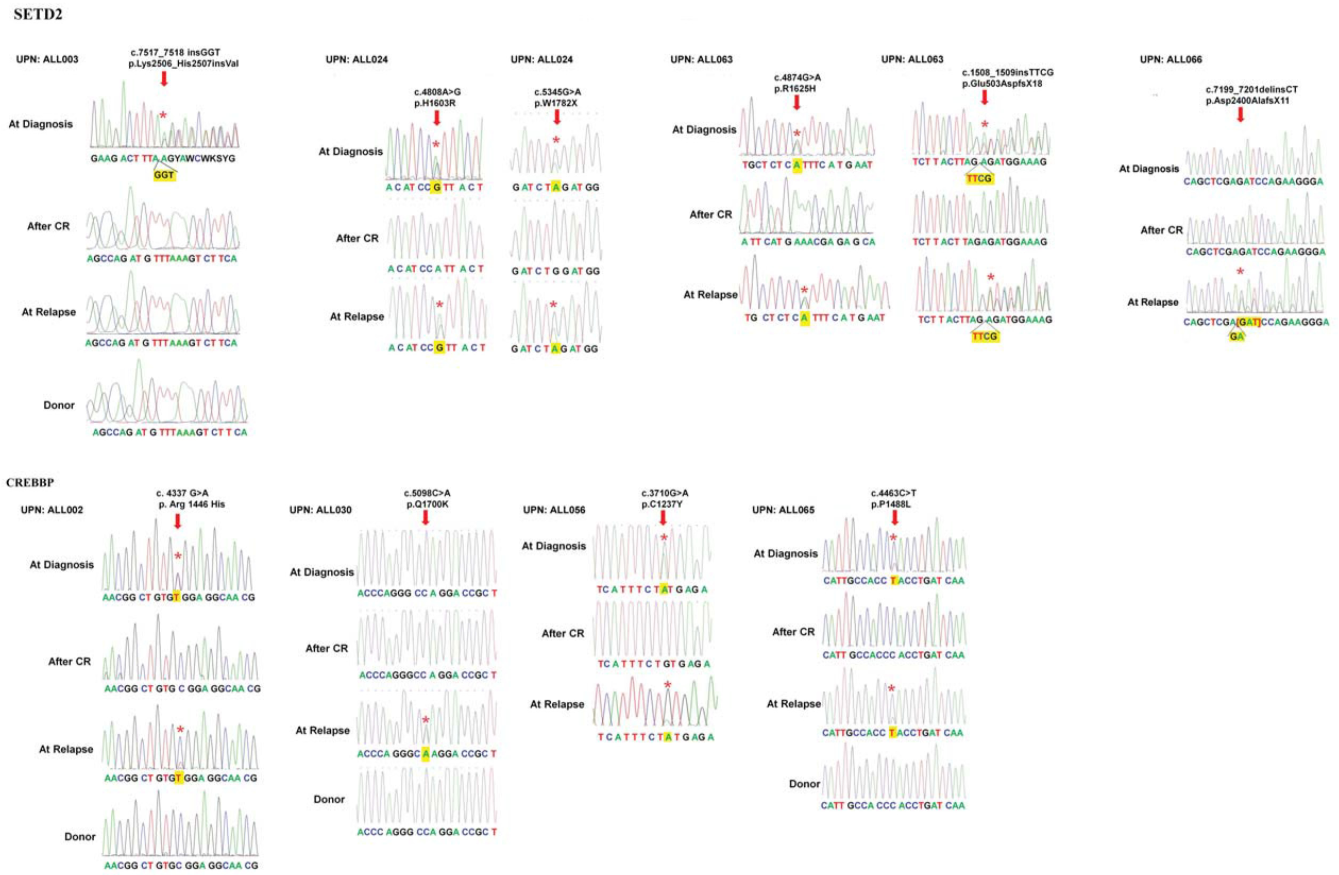
SETD2
UPN: ALL003
c.7517_7518 insGGT
p.Lys2506_His2507insVal
At Diagnosis
After CR
At Relapse
Donor
UPN: ALL024
c.4808A>G
p.H1603R
UPN: ALL024
c.5345G>A
p.W1782X
UPN: ALL063
c.4874G>A
p.R1625H
UPN: ALL063
c.1508_1509insTTCG
p.Glu503AspfsX18
UPN: ALL066
c.7199_7201delinsCT
p.Asp2400AlafsX11
CREBBP
UPN: ALL002
c. 4337 G>A
p. Arg 1446 His
UPN: ALL030
c.5098C>A
p.Q1700K
UPN: ALL056
c.3710G>A
p.C1237Y
UPN: ALL065
c.4463C>T
p.P1488L

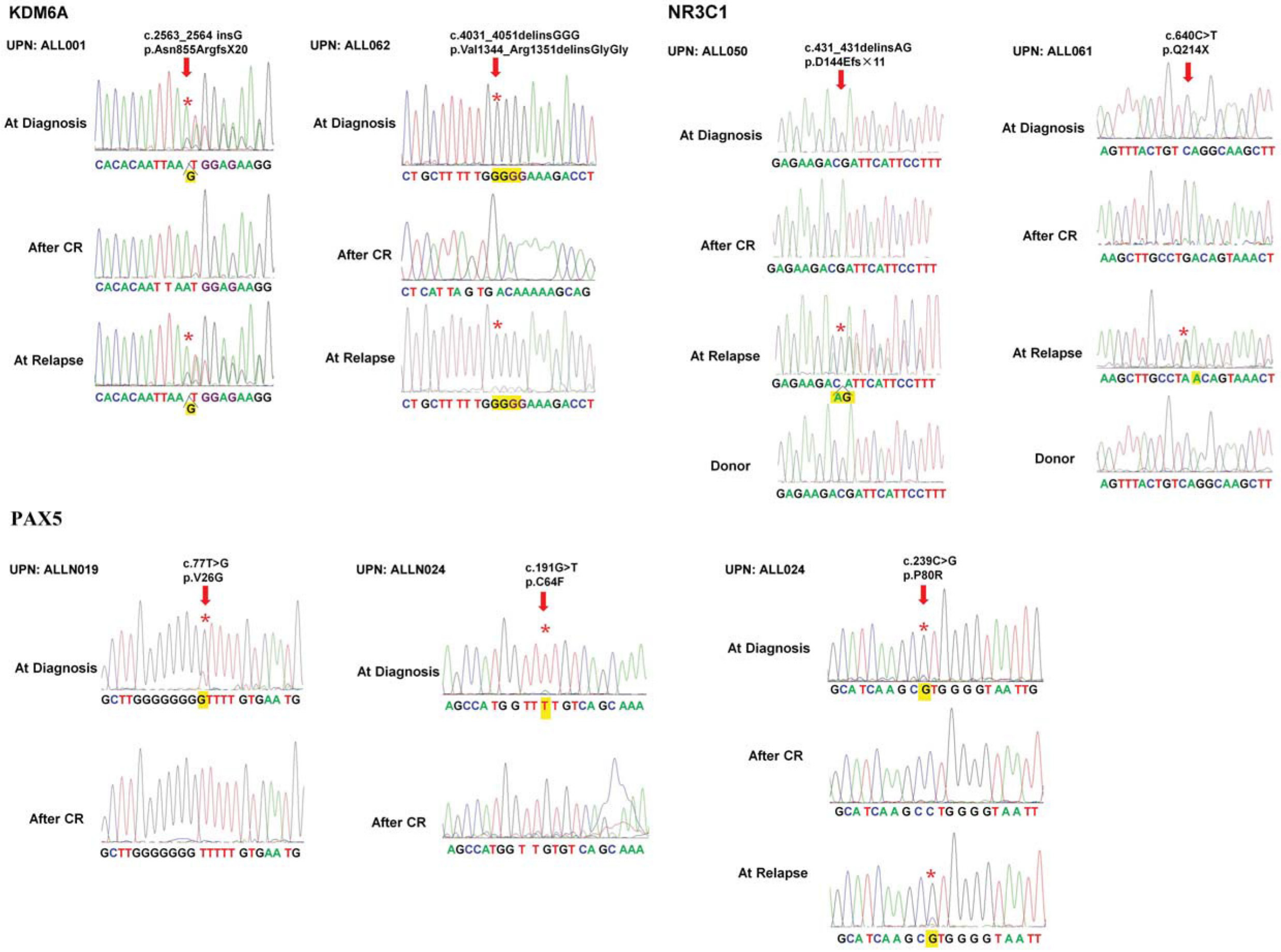

Supplementary Figure S2–S3: Sequencing results of 5 mutated epigenetic regulators *(SETD2*, CREBBP*, KDM6A, NR3C1* and *PAX5)* in Ph⁻ adult B-ALL. Distinct mutations and amino acid replacements from matched DNA samples at diagnosis, control samples collected during CR and at relapse as well as respective donor samples (if necessary), were confirmed by Sanger sequencing. (*Sequencing result of SETD2-ALL003- At Diagnosis was indicated sequence of respective complementary chains. *Sequencing results of CREBBP-ALL002, were indicated sequences of respective complementary chains).

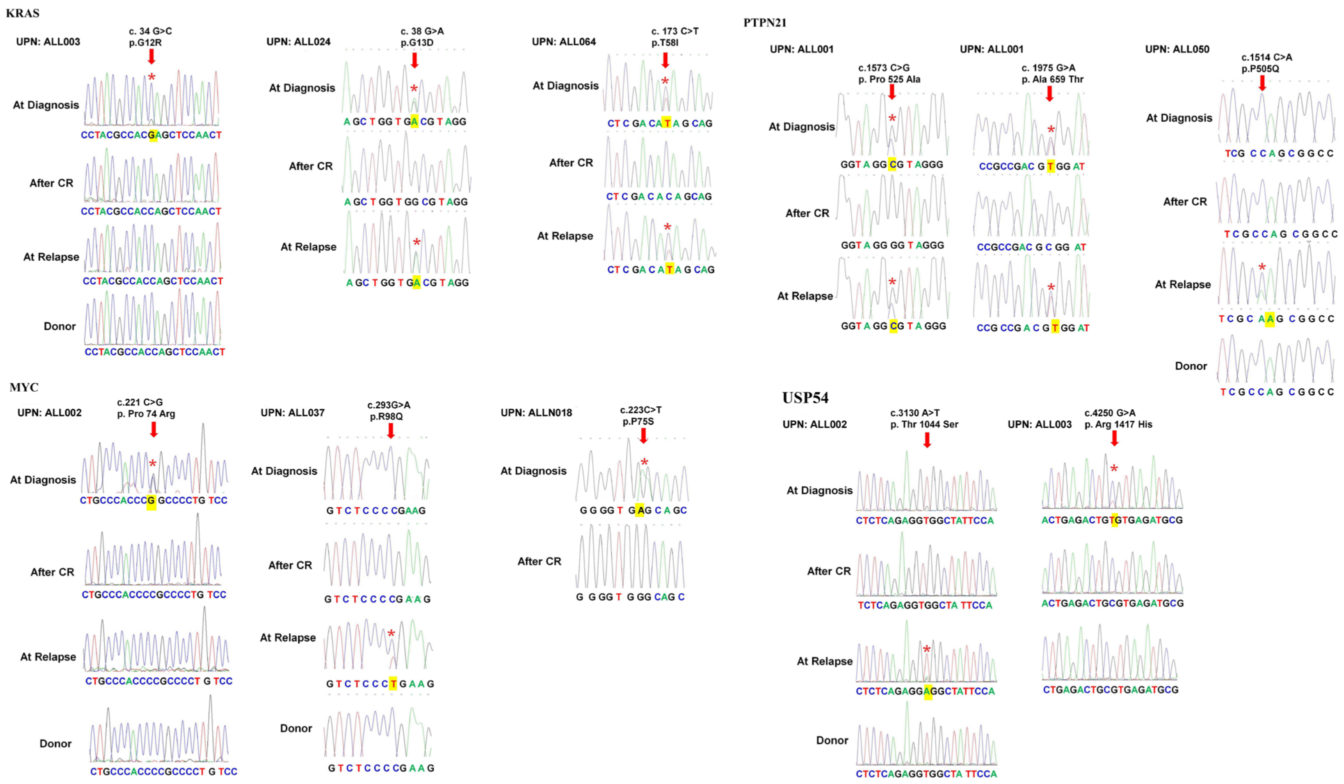

Supplementary Figure S4: Sequencing results of signaling factors *(KRAS, PTPN21*, MYC*, USP54)* in adult Ph⁻ B-ALL. Distinct mutations and amino acid replacements from matched DNA samples at diagnosis, control samples collected during CR and at relapse as well as respective donor samples (if necessary), were confirmed by Sanger sequencing. (*Sequencing results of PTPN21-ALL001, MYC-ALL037, MYC-ALLN018, USP54-ALL002 and USP54-ALL003 were indicated sequences of respective complementary chains).

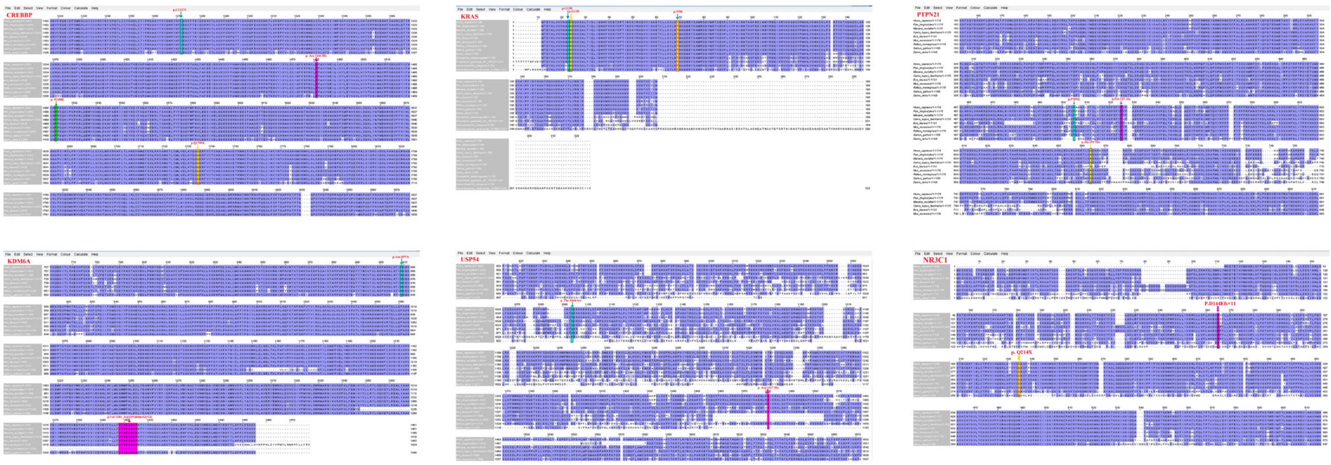

Supplementary Figure S5: Mutated domains in 6 mutated genes *(CREBBP, KRAS, PTPN21, KDM6A, USP54* and *NR3C1)* are highly conserved across species. The mutation sites are marked in red and indicated with arrow.

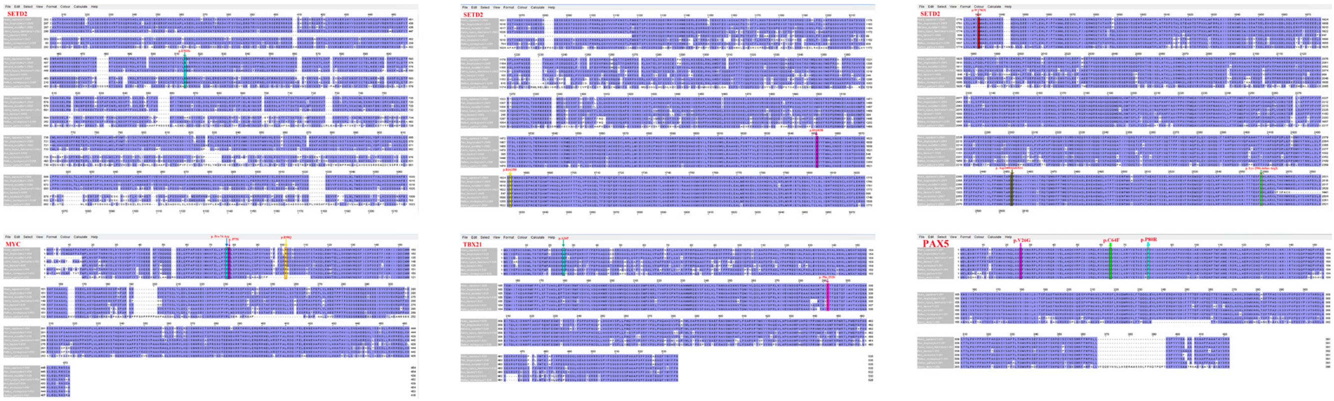

Supplementary Figure S6: Mutated domains in 4 mutated genes *(SETD2, MYC, TBX21* and *PAX5)* are highly conserved across species. The mutation sites are marked in red and indicated with arrow.
